# *TMPRSS6* rs855791 Polymorphism Status in Children with Celiac Disease and Anemia

**DOI:** 10.3390/nu13082782

**Published:** 2021-08-13

**Authors:** Klaudia Urbaszek, Natalia Drabińska, Anna Szaflarska-Popławska, Elżbieta Jarocka-Cyrta

**Affiliations:** 1 Department of Pediatrics, Gastroenterology and Nutrition, Faculty of Medicine, Collegium Medicum, University of Warmia and Mazury, Żołnierska 18A Str., 10-561 Olsztyn, Poland; klaudiaferus@o2.pl; 2 Institute of Animal Reproduction and Food Research of Polish Academy of Sciences, Tuwima 10 Str., 10-748 Olsztyn, Poland; n.drabinska@pan.olsztyn.pl; 3 Laboratory for Pediatric Endoscopy and Gastrointestinal Function Testing, Ludwik Rydygier Collegium Medicum in Bydgoszcz, Nicolaus Copernicus University of Torun, Jagiellonska 13-15, 85-067 Bydgoszcz, Poland; aszaflarska@wp.pl

**Keywords:** celiac disease, anemia, iron metabolism, gluten-free diet, rs855791, *TMPRSS6*

## Abstract

Celiac disease (CD) is an autoimmune chronic inflammatory disease occurring in genetically predisposed individuals in response to the intake of gluten. Clinical presentation can be heterogeneous. Iron-deficient anemia (IDA) is one of the most common extra-intestinal manifestations of CD. Although IDA usually reverts with a gluten-free diet (GFD), some patients show persistent IDA, the mechanisms of which are poorly understood. Recent studies suggest an association between the rs855791 polymorphism in the *TMPRSS6* gene and persistent IDA in adults with CD. The current study aimed to assess the potential link between rs855791 and persistent IDA in pediatric patients with CD. The study included 106 children diagnosed with CD between 2015 and 2019. Clinical and blood parameters (including blood count, serum iron) were collected at diagnosis and after ≥12 months of GFD, and the rs855791 genotype was assessed for each patient. IDA was present at diagnosis in 25 patients (23.6%); only three (3%) had persistent IDA after GFD. The prevalence of rs855791 genotypes was 9% (*n* = 10) for TT, 53% (*n* = 56) for CT, and 38% (*n* = 40) for CC. There was a tendency toward a higher proportion of the T allele in patients with IDA and lower hemoglobin in the TT genotype but without statistical significance. An association between rs855791 and persistent IDA was not observed. These findings suggest that persistent IDA is uncommon in pediatric patients with CD. The prevalence of rs855791 in children with CD is reported for the first time.

## 1. Introduction

Celiac disease (CD) is an autoimmune chronic inflammatory disease that occurs in genetically predisposed individuals in response to gluten intake [1]. The hallmarks of CD include the presence of auto-antibodies directed against tissue transglutaminase 2 (TTG) and small intestinal enteropathy characterized by villous atrophy, crypt hyperplasia and lymphocytic infiltration of the epithelium. The only therapy for CD is a lifelong gluten-free diet (GFD).

The clinical presentation of CD is heterogeneous. The classical form is characterized by chronic diarrhea and failure to thrive. In non-classical CD, diarrhea is absent; instead, gastrointestinal symptoms may include abdominal pain, bloating, nausea, and constipation. Importantly, both classical and non-classical CD can also be accompanied by a wide range of extra-intestinal symptoms. Iron-deficient anemia (IDA), characterized by microcytosis and low serum iron and ferritin levels, is one of the most common extra-intestinal manifestations of CD, irrespective of patients’ age and sex [2]. IDA has been reported in 12% to 69% of cases of newly diagnosed CD [2,3,4]. On the other hand, one in 31 patients with IDA has histologic evidence of CD [5].

Iron deficiency can result from chronic blood loss, inadequate dietary iron intake, impaired iron absorption, and/or chronic inflammation. The latter two factors, in particular, have been incriminated as potential causes of IDA in patients with CD. A gluten-free diet limits intestinal inflammation, normalizes gut epithelium and restores the absorption of nutrients [6]. However, persistent IDA has been reported in 15% to 28% of adult patients with CD and in 16% of children, despite adherence to GFD [7,8]. The mechanisms responsible for the persistence of anemia and iron deficiency in CD have not yet been elucidated [7,8].

Hepcidin, a key player in iron metabolism, can decrease iron levels by inhibiting intestinal iron absorption and iron release by macrophages and hepatocytes [9,10]. The regulation of hepcidin synthesis is a complex process. Transmembrane Serine Protease 6 (TMPRSS6), also known as Matriptase-2, is a liver-specific enzyme that negatively regulates hepcidin production [11]. Interestingly, several studies have linked the rs85579 single nucleotide polymorphism (SNP) in the *TMPRSS6* gene to hemoglobin levels and/or iron status in large cohorts of healthy adults and adults with CD or with IDA [12,13,14]. Nevertheless, the potential association between *TMPRSS6* polymorphisms and anemia in children with CD has not been addressed to date.

The aim of this study was to investigate the association between the rs855791 *TMPRSS6* polymorphism, anemia and iron status in children with CD at diagnosis and after treatment with GFD, and to explore the potential link between rs855791 and persistent IDA in this population.

## 2. Materials and Methods

### 2.1. Study Design and Population

The study was carried out between October 2018 and August 2020 in the Provincial Specialist Children’s Hospital in Olsztyn and the Department of Paediatrics, University Hospital No.1 in Bydgoszcz, Poland. Using patient medical records, patients diagnosed with CD were retrospectively identified in both centers between January 2015 and December 2018 and prospectively enrolled patients diagnosed with CD during 2019. Children were diagnosed with CD if they fulfilled the 2012 ESPGHAN criteria: positive IgA TTG or anti-endomysial antibodies (EMA) and biopsy-proven enteropathy according to Marsh criteria [15]. Patients with concomitant inflammatory bowel disease or with Down syndrome were excluded. In all patients, clinical symptoms, anthropometric data, blood parameters, serum iron levels and serum TTGlevels at diagnosis were retrieved retrospectively from medical records. The presence of concomitant-associated conditions, such as type 1 diabetes mellitus or autoimmune thyroid disease, was also recorded. Children with CD were then subdivided into those with IDA and those without anemia at diagnosis, with anemia being defined based on hemoglobin (HGB) levels according to age and gender using the World Health Organization (WHO) criteria [16] (Supplementary Table S1). These two groups were compared in terms of clinical characteristics, histology, hematological and biochemical parameters, adherence and response to GFD.

Patients were followed at their respective gastroenterology outpatient clinics. A follow-up visit was scheduled for each patient as part of this study after at least 12 months of GFD. Hematologic response to GFD was defined as normalization of HGB levels based on WHO criteria. Adherence to GFD was assessed based on parents’/patients’ reports and on normalization or a significant decrease in anti-TTG antibodies.

### 2.2. Ethics

Parents and caregivers of all study participants were informed about the potential benefits and risks and signed a written consent form. A consent form was also signed by patients aged 16 years or above. The experimental design and all procedures were approved by the Bioethics Committee of the Faculty of Medical Sciences of the University of Warmia and Mazury in Olsztyn (permission No. 32/2017 granted on 22 June 2017).

### 2.3. Sample Collection

At the follow-up visit, blood samples were collected for blood count, C-Reactive Protein (CRP), serum ferritin, iron, hepcidin and Interleukin-6 (IL-6) levels, and for genomic testing for the *TMPRSS6* rs855791 polymorphism. Peripheral blood samples (2 mL) and serum samples (2 mL) were collected from all patients and stored at −80 °C until the biochemical and genetic analyses.

### 2.4. DNA Analysis

Genomic DNA was extracted from 500 µL of total peripheral blood stabilized with EDTA using an InnuPREP Blood DNA Midi Kit and Smart Blood DNA Midi Kit (cat. No AJ845-KS-1030050, cat. no 845-KS-8100050, Analytik Jena AG, Jena, Germany), according to the manufacturer’s instructions. The quality and quantity of extracted DNA were estimated using a NanoDrop 1000 spectrophotometer (Thermo Fisher Scientific, Waltham, MA, USA).

The SNP genotyping analysis was performed in a final volume of 10 μL, using 3 μL of DNA, 0.5 μL of specific primers with probes (TaqMan SNP Genotyping Assay ID: C_32899902_10), Context Sequence [VIC/FAM]: GCGTGGCGTCACCTGGTAGCGATAG[A/G]CCTCGCTGCACAGGTCCTGTGGGAT, and TaqPath™ ProAmp™ Master Mix (cat. No A30865, Applied Biosystem, Waltham, MA, USA). Real-time PCR was carried out on a ViiA™ 7 Real-Time PCR System (Life Technologies) under default thermal cycling conditions. Amplification was performed with pre-read step for 30 s at 60 °C; next, an initial denaturation step for 5 min at 95 °C, followed by 45 cycles of 15 s at 95 °C and 60 s at 60 °C. The last step was the post-read step for 30 s at 60 °C. Polymerase chain reactions were performed in duplicate and on negative controls prepared using water instead of the DNA template. The results of *TMPRSS6* genotype and allele frequencies were analyzed using GraphPad Prism 8.4 software (GraphPad Software, Inc., San Diego, CA, USA) using Fisher’s exact test.

Hereafter, considering that the rs855791 polymorphism site represents the c.2207 position of the *TMPRSS6* coding sequence based on RefSeq NM_153609.3, thymine at this position (resulting in p.Val736) is designated as the “T” allele, and a cytosine (resulting in p.Ala736) is defined as the “C” allele.

### 2.5. Serum Biochemical Analyses

The concentration of hepcidin in serum samples was measured using a commercial ELISA kit (FineTest, Wuhan, China). The detection range of the test was 15.625–1000 pg/mL, the sensitivity was 9.375 pg/mL, and the coefficient of variation was below 10%.

The serum concentration of IL-6 was measured using Human IL-6 ELISA Kit (FineTest, Wuhan, China). The detection range of the test was 4.688–300 pg/mL, the sensitivity was 2.813 pg/mL, and the coefficient of variation was below 10%.

ELISA kits were used according to manufacturers’ protocols. All ELISAs were analyzed using a Biochrom^®^ Asys UVM 340 Microplate Reader (Biochrom Ltd., Cambridge, UK).

### 2.6. Statistical Analysis

All statistical analyses were performed using the Statistica 12 (StatSoft, Tulsa, OK, USA) and GraphPad Prism version 8.0.0 for Windows (San Diego, CA, USA) software. The normality of quantitative variables was tested with the Shapiro–Wilk W test. Differences in characteristics between the genotypes were tested with the parametric ANOVA test or the non-parametric Kruskal–Wallis test, as appropriate. A comparison between the results at diagnosis and after 12 months of GFD was analyzed using Student’s t-test for dependent variables or Wilcoxon signed-rank tests, as appropriate, according to the normality, considering the significance at three levels: (*) = *p*-value < 0.05; (**) = *p*-value < 0.01; (***) = *p*-value < 0.001. Correlations between parameters were analyzed using a Spearman’s rank correlation coefficient test.

## 3. Results

During the study period, 128 children with CD were enrolled and the results of 106 children, including 62 girls (58.5%) and 44 boys (41.5%), were used for the analysis. Twenty-two participants have been removed because of the lack of blood test results in the enrollment or follow-up visit. All patients were treated for at least 12 months with GFD and vitamin D3 supplementation. There was no statistically significant difference in GFD duration between the anemic and non-anemic groups (mean, 29.9 and 30.0 months, respectively). Clinical characteristics and the rs855791 polymorphism status of CD patients with and without IDA at diagnosis are shown in Table 1.

At the time of diagnosis, sixty-six patients (62%) presented gastrointestinal symptoms (e.g., abdominal pain, diarrhea and/or constipation), and 22 (21%) had weight and/or height deficiency. In 17 cases (16%), CD co-occurred with type 1 diabetes. IDA was present at diagnosis in 25 patients (23.6%). Of those, ten patients had mild anemia, fifteen had moderate anemia, and three had severe anemia, according to WHO classification (Supplementary Table S1).

**Figure 1:**
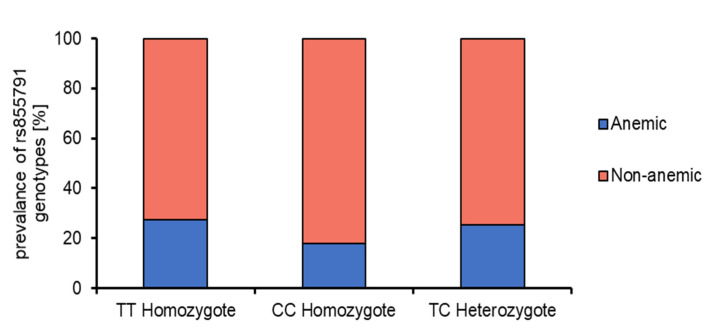
Proportion of the frequency between the polymorphism varieties in anemic and non-anemic children with celiac disease.

There was a tendency toward a higher proportion of the T allele in patients with IDA at diagnosis than in those without IDA (40% vs 34.6%, respectively), but this difference did not reach statistical significance (Table 1, Figure 1).

Hematological and biochemical parameters at diagnosis in patients stratified according to the rs855791 polymorphism status are presented in Table 2. No statistically significant differences in blood parameters at diagnosis were observed between the three rs855791 genotypes (i.e. homozygous TT, heterozygous CT, and homozygous CC), although there was a tendency toward lower HGB levels in the TT genotype and higher HGB levels in the CC genotype (median HGB = 11.95 g/dL, 12.60 g/dL, and 13.10 g/dL for homozygous TT, heterozygous CT, and homozygous CC, respectively). When each genotype group was further subdivided into children with or without anemia, significant differences between anemic and non-anemic children were observed in terms of blood parameters: HGB, hematocrit (HCT), mean corpuscular volume (MCV), mean corpuscular hemoglobin (MCH), as expected, but no significant differences in those parameters were found between the genotypes. However, iron serum levels were significantly lower in anemic children with the CC genotype (median, 23 µg/dL) than in anemic children with the other two genotypes (median, 56 µg/dL for the TT genotype and 36 µg/dL for the CT genotype).

Hematological and biochemical parameters after 12 months of GFD in patients stratified according to the rs855791 polymorphism status are shown in Table 3. A significant reduction in TTG levels as compared to levels at diagnosis supported good dietary adherence in all subgroups. In general, twenty-two of the 25 patients (88%) who initially showed IDA had normalized their HGB levels after 12 months of GFD. In three children (12%), anemia persisted despite 12 months GFD, although its severity decreased. The rs855791genotypes of these three patients included: one patient with genotype TT and two with CT. Finally, two further children who did not have IDA at diagnosis developed anemia during 12 months of GFD (these cases were not considered as “persistent IDA” in the study).

Hematological parameters in all children with anemia at diagnosis significantly improved on GFD. In non-anemic children with a homozygous CC genotype, these parameters also increased after GFD, which was not the case for the other two genotypes.

**Figure 2:**
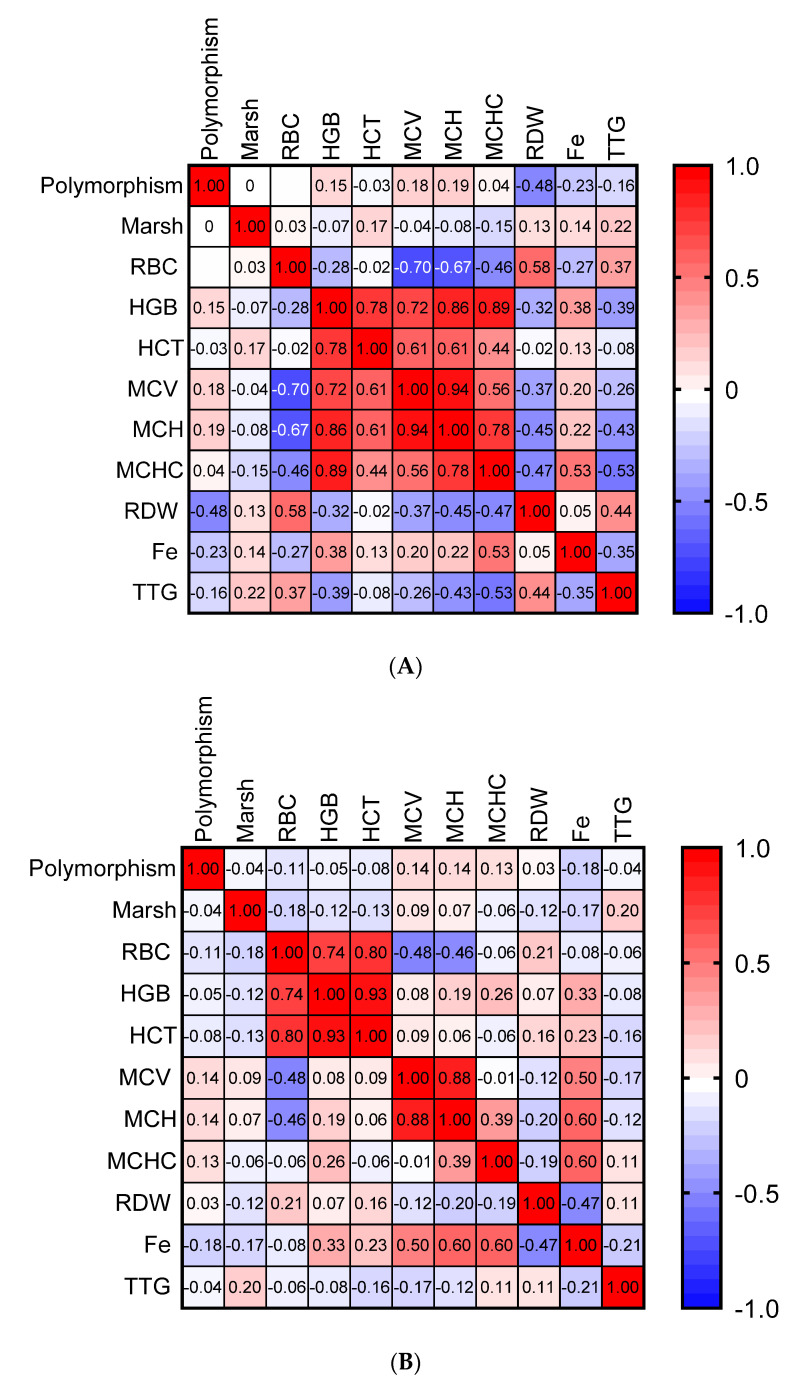
Heatmap representation of the correlations between anemia-related parameters at CD diagnosis in children with (**A**) and without (**B**) anemia. Marsh—Marsh classification; RBC—red blood cells; HGB—hemoglobin; HCT—hematocrit; MCV—mean corpuscular volume; MCH—mean corpuscular hemoglobin; MCHC—mean corpuscular hemoglobin concentration; RDW—red cell distribution width; Fe—iron; TTG—auto-antibodies directed against tissue transglutaminase 2.

**Figure 3:**
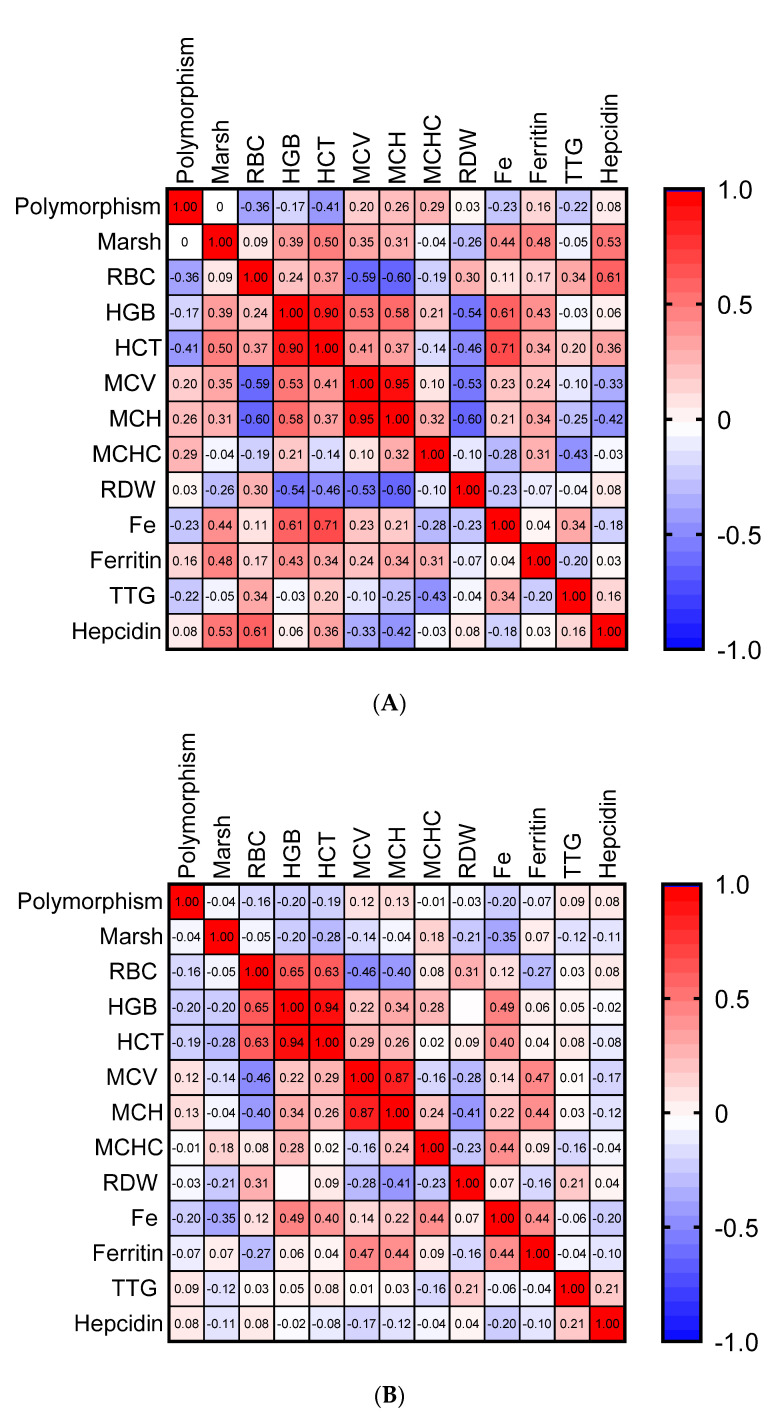
Heatmap representation of the correlations between anemia-related parameters after 12 months of GFD in children with (**A**) and without (**B**) initial anemia. Marsh—Marsh classification; RBC—red blood cells; HGB—hemoglobin; HCT—hematocrit; MCV—mean corpuscular volume; MCH—mean corpuscular hemoglobin; MCHC—mean corpuscular hemoglobin concentration; RDW—red cell distribution width; Fe—iron; TTG—auto-antibodies directed against tissue transglutaminase 2.

A correlation analyses between (Spearman’s rank correlation coefficient test) at diagnosis (Figure 2) and after ≥12 months of GFD were performed to evaluate the association between the parameters (Figure 3). The rs855791 polymorphism status was not significantly correlated with any parameters when all patients were analyzed together (not shown). However, several correlations were observed when the anemic and non-anemic groups were analyzed separately (Figure 2 and Figure 3).

At diagnosis, in the anemic group (Figure 2A), a moderate negative correlation between the CT genotype and positive between TT and red cell distribution width (RDW) was observed (r = −0.48, *p* = 0.018), which was not seen in non-anemic children (Figure 2B). As expected, significant positive correlations were also observed between HGB, HCT, MCV, mean corpuscular hemoglobin concentration (MCHC) and MCH (in particular in the anemic group), and between iron concentration, HGB and MCHC (in both groups). Conversely, a negative correlation between red blood cells (RBC), MCV, and MCH was observed in both groups.

**Figure 4:**
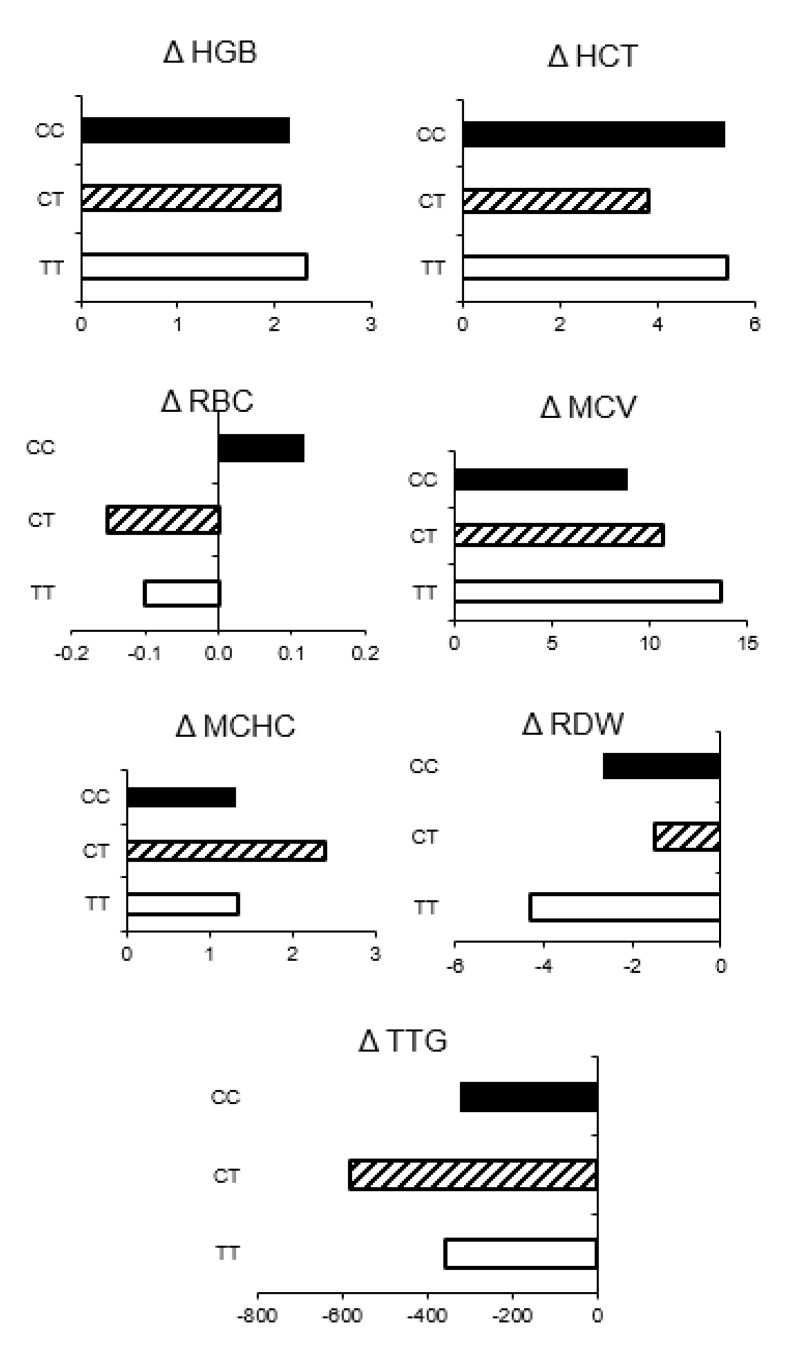
Changes in morphometric parameters and TTG levels between values at diagnosis and after 12 months of GFD in children with CD and initial anemia (*n* = 25), according to the genotype. The results are expressed as the mean of delta values.

After 12 months of GFD (Figure 3A), in the anemic group, a correlation between the TT genotype and HCT was close to the significance threshold (r = −0.4, *p* = 0.06). A statistically significant negative correlation between hepcidin and MCV and MCH was also noted in this group (MCV: r = 0.53, *p* = 0.02; MCH: r = 0.61, *p* = 0.01). In the non-anemic group, no significant correlations between polymorphism status and other parameters (or between hepcidin levels and other parameters) were observed after 12-months of GFD (Figure 3B). Figure 4 shows a graphical representation of the range of changes in iron metabolism parameters and in TTG levels between diagnosis and the follow-up visit after 12 months of GFD in children with CD and initial anemia, stratified by genotype, calculated as an average change (delta) for the individual patients. The trend of changes for most parameters was similar for all genotypes, except for RBC, in which an increase was noted in the CC genotype, while in homozygous TT and heterozygous CT, a decrease was observed.

## 4. Discussion

Celiac disease (CD) is a lifelong disorder characterized by a heterogeneous clinical picture with both gastrointestinal and extra-intestinal symptoms and can affect individuals of any age [17]. In recent decades, a shift in terms of frequency of diagnosis from the classical form of CD, characterized by malabsorption, to non-classical forms has been reported. The increasing number of recognized atypical and asymptomatic cases could be explained at least in part by screening at-risk groups and by higher awareness of various clinical presentations of CD [18]. Anemia is one of the most common extra-intestinal symptoms of CD. It can co-occur with other symptoms or even be the only manifestation of the disease [19]. The reported prevalence of iron deficiency and IDA in CD varies between studies, depending on study design, population and patient age [20].

In the current study, IDA at diagnosis was present in 23.6% of cases, which is a prevalence similar to what has been observed in other pediatric studies. In a study in Finland by Nurminen et al. [21], anemia was the second most common extra-intestinal symptom after poor growth in children with CD and it was present in 18% of cases. In another study of preschool children, iron deficiency and growth retardation were the second most common symptom of CD, after abdominal pain [22]. In a recent study of 653 children in Central Europe, the reported prevalence of IDA in participating countries ranged from 4.3% to 24.9% [18]. Nevertheless, some other studies in children reported lower (9%) [23] or higher (84%) [24] frequencies.

Enteropathy is thought to be the main factor responsible for diminished iron uptake in the intestine in CD. The only treatment for CD is a GFD, which results in normalization of the intestinal mucosa and clinical improvement of gastrointestinal and extra-intestinal symptoms [6,25]. However, in a subset of patients, IDA persists despite adherence to GFD [7]. The reported prevalence of persistent IDA in adults with CD ranges from 5% to as high as 45.5% [8,13,26].

Conversely, in the current study, only three of the 25 pediatric patients with CD who had IDA at diagnosis showed persistent anemia after 12 months of GFD, accounting for 2.8% of the entire cohort (*n* = 106). Overall, the data suggest that persistent IDA is not a common complication of CD in pediatric patients. This is in line with a study by Wessels et al. [23], who reported persistent anemia in only 1% of children with CD, although a higher rate of persistent IDA (16%) was observed by other authors [22,26].

One potential explanation for this difference between pediatric and adult patients is that children are usually diagnosed earlier in the disease. As such, the delay in diagnosis in adults can result in more advanced villous atrophy at diagnosis, more profound iron deficiency and, consequently, more severe anemia [7]. Thus, even prolonged adherence to a GFD without iron supplementation may not be enough to normalize the anemia in adult patients. While IDA in CD has been linked to histopathological changes in the small intestine resulting in iron malabsorption [21], additional genetic factors may contribute to anemia at diagnosis and/or persistent anemia in CD patients. In 2011, two genome-wide association (GWAS) studies identified an association between the rs855791 non-synonymous coding SNP located in the *TMPRSS6* locus and hemoglobin levels and iron status in large populations [12,27]. The *TMPRSS6* protein (Matriptase-2) is a liver-specific type II plasma membrane serine protease, which can negatively regulate hepcidin production by modulating downstream signaling pathways involved in hepcidin gene expression [11]. The expression of hepcidin is induced by high iron stores or inflammation. In turn, hepcidin decreases iron levels by blocking intestinal iron absorption at the basolateral membrane of enterocytes and inhibiting iron release from hepatocytes and macrophages [10]. Thus, the repression of hepcidin by *TMPRSS6* could facilitate iron absorption and potentially promote erythropoiesis. Conversely, *TMPRSS6* loss of function may result in unregulated hepcidin synthesis, reduced iron absorption, and iron-deficiency anemia [28].

**Figure 5:**
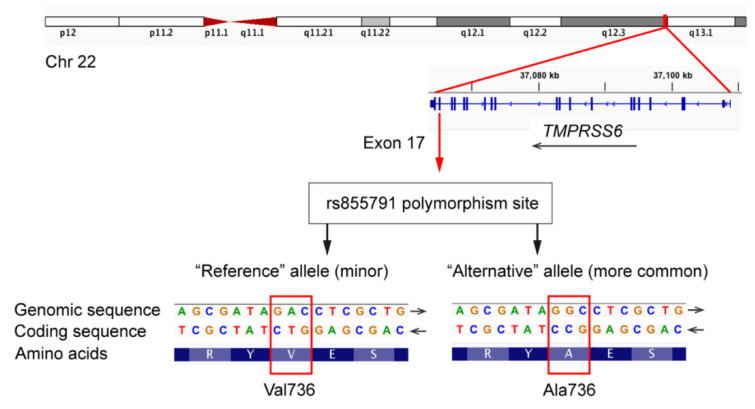
Schematic representation of the rs855791 polymorphism.

The rs85579 non-synonymous coding SNP causes an alanine-to-valine change (p.A736V) in the catalytic domain of the *TMPRSS6* protein (Figure 5). The “T allele” (also referred to as the “A allele” in some studies if using the genomic DNA annotation), results in a valine at the 736aa position, and the “C allele” (also referred to as the “G allele” using the genomic DNA annotation) results in an alanine at the 736aa position [12,27]. The two above-cited GWAS studies found the T allele to be associated with a lower blood HGB concentration per copy of the T allele [12] and lower serum iron levels, transferrin saturation, and MCV [29]. These results were subsequently supported by other studies, whereby the T allele was found to be associated with lower HGB and serum iron levels in Chinese women [30], and the C allele was associated with lower hepcidin and higher iron parameters in the genetically isolated “Val Borbera” population in Italy [31]. *In vitro* experiments provided a functional rationale by showing that the Ala736 form of the *TMPRSS6* protein inhibits hepcidin expression more effectively than the Val736 form. According to available data, the prevalence of rs855791 alleles is heterogenous in different populations. The T allele is the less frequent allele, with a frequency of 0.50 in the white population [29]. In a study by Chambers et al., the TT genotype was presented in 19% of individuals of European ancestry and in 27% of individuals of Indian Asia ancestry [12].

The current study is the first to report the prevalence of the rs855791 polymorphism in children with CD. Of note, although the T allele is the “reference” allele in the hg38 and hg19 human reference genomes (Figure 5), it was found to be less frequent than the C allele: the overall allele frequencies across the study cohort (*n* = 106) were 35.8% for the T allele and 64.2% for the C allele, respectively. This is in agreement with the numbers reported in previous studies [13,14]. Thus, the T allele appears to be a “minor reference allele”, which is a known phenomenon in genomic data analysis [32]. 

The literature contains only two studies addressing the link between rs855791 SNP and anemia in patients with CD. Elli et al. [14] evaluated the association between the rs855791 variant and persistent IDA in 38 adult patients with CD treated by GFD. They found the prevalence of the rs855791 SNP to be significantly higher in adult patients with CD than in the control group of non-CD adults (87% vs. 62%, respectively). Persistent anemia was seen in 16 patients (42%). There was no significant difference in the prevalence of the rs855791 polymorphism between CD adults with persistent IDA and those without IDA. Nevertheless, associations between iron status, hemoglobin and hepcidin levels and the polymorphism status were not evaluated. De Falco et al. [13] also studied the rs855791 polymorphism in adult patients with CD and found that the T allele was significantly more frequent among patients with persistent IDA, and it was associated with a lower response of hematological and iron parameters to oral iron supplementation in patients with persistent IDA.

Contrary to these results, the current study did not find a significant association between the T allele and the persistence of anemia in children with CD who presented IDA at diagnosis (*n* = 25). This could be due to the low number of patients with persistent IDA in the current study (3%), as compared to 45.4% of adult anemic patients in the study by De Falco et al. [13]. A tendency was observed toward a higher prevalence of the T allele in CD children with IDA at diagnosis than in those without IDA (40% vs. 34.6%, respectively) and toward lower HGB levels in children with the TT genotype. However, these differences did not reach statistical significance, which was possibly due to the small sample size, and they warrant further investigation in larger studies.

Although genetic factors in anemia in CD have gained much scientific attention, data remain very limited in pediatric patients. One other study found the homozygous intronic IVS4 + 44C > A polymorphism in the DMT1 gene associated with a four-fold risk of developing anemia in children with CD, regardless of the degree of villous atrophy [33]. Additional studies in large patient cohorts are needed to confirm these findings.

As far as it is known, this is the first study to analyze the associations between a *TMPRSS6* polymorphism and anemia in the pediatric population with CD. The main strength of this study is the homogenous nature of the cohort in terms of diagnostic robustness, as the diagnosis was confirmed by histology and immunological markers in all cases. Another important point is the wide range of clinically relevant parameters reported herein, including inflammatory markers and hepcidin levels analyzed in the current study. The principal limitations include the relatively small group of patients, the absence of a control group and the fact that data at diagnosis were collected retrospectively (as a consequence, information on all iron metabolism parameters and symptoms onset to diagnosis was not always available).

## 5. Conclusions

The current study of 106 children with CD suggests that persistent iron-deficiency anemia (IDA) is not a common finding in pediatric patients with CD. IDA was present at diagnosis in 23.6% of children in the study and normalized on a GFD without iron supplementation in most (88%) patients. Significant differences in the prevalence of the *TMPRSS6* rs855791 polymorphism were not observed between CD children with or without anemia, although a tendency toward a higher proportion of the T allele in patients with IDA was noted. Contrary to what has been reported in adults, the rs855791 polymorphism was not found to be a predicting factor of persistent anemia in children with CD.

## Figures and Tables

**Table 1:** Characteristics of CD patients with and without IDA at diagnosis and *TMPRSS6* gene polymorphism status.

Parameter	CD with IDA	CD without IDA
Number	25 23.6%	81 76.4%
Gender		
Girls *n*.	20 80.0%	42 51.9%
Boys *n*.	5 20.0%	39 48.1%
Age [years]	8.06 ± 4.89	8.04 ± 4.12
Height [m]	1.23 ± 0.29	1.28 ± 0.24
Weight [kg]	28.66 ± 19.43	28.10 ± 15.38
BMI [kg/m^2^]	16.99 ± 3.97	16.18 ± 2.82
Main complaints		
Gastrointestinal *n*. * Classical Non-Classical	15 60.0% 11 73.3% 4 26.7%	51 62.9% 29 56.9% 22 43.1%
Extraintestinal *n*. **	25/3 ^1^ 100.0%/12.0%	62 76.5%
Diabetes type 1 *n*.	0.0 0.0%	17 20.9%
Polymorphism of rs855791		
TT homozygote *n*.	3 12.0%	7 8.6%
CT heterozygote *n*.	14 56.0%	42 51.9%
CC homozygote *n*.	8 32.0%	32 39.5%
Allele frequency		
Allele T *n*.	20 40.0%	56 34.6%
Allele C *n*.	30 60.0%	106 65.4%

Abbreviations: BMI, body mass index; *n*. number; Values are presented as mean ± standard deviation (SD), *, e.g. abdominal pain, diarrhea, constipation, weight loss, **, e.g. weight and height deficiency, osteoporosis, anemia, ^1^ Three patients presented anemia as the only symptom.

**Table 2:** Hematological characteristics in CD patients at the time of diagnosis according to the genotype. Data are expressed as a median (P25–P75).

Parameter	TT Homozygote		CC Homozygote		CT Heterozygote	
N	10		40		56	
RBC [×10^6^/mm^3^]	4.75 (4.53–4.95) ^a 1^		4.70 (4.43–5.08) ^a^		4.70 (4.40–4.93) ^a^	
HGB [g/dL]	11.95 (10.78–13.05) ^a^		13.10 (11.68–13.63) ^a^		12.60 (11.30–13.30) ^a^	
HCT [%]	37.80 (34.40–40.40) ^a^		38.80 (35.05–40.80) ^a^		37.30 (34.35–40.10) ^a^	
MCV [µm^3^]	79.00 (75.00–82.25) ^a^		81.00 (77.50–86.00) ^a^		82.00 (79.00–86.00) ^a^	
MCH [pg]	26.05 (23.13–26.40) ^a^		27.40 (25.85–28.70) ^a^		27.30 (26.15–28.45) ^a^	
MCHC [g/dL]	32.50 (31.90–32.80) ^a^		32.90 (32.65–33.80) ^a^		33.10 (32.30–33.80) ^a^	
RDW [%]	12.60 (12.50–14.30) ^a^		13.40 (12.60–15.48) ^a^		13.20 (12.60–14.20) ^a^	
Fe [µg/dL]	59.00 (56.00–84.00) ^a^		41.00 (26.50–107.50) ^a^		54.00 (41.25–86.00) ^a^	
TTG [U/mL]	289.5 (112.8–398.5) ^a^		201.0 (49.40–801.0) ^a^		191.0 (41.10–539.0) ^a^	
	Anemic	Non-anemic	Anemic	Non-anemic	Anemic	Non-anemic
N	3	7	8	32	14	42
RBC [× 10^6^/mm^3^]	4.70 (4.70–4.70) ^a^	4.80 (4.45–5.00) ^a^	4.80 (4.45–4.95) ^a^	4.70 (4.43–5.08) ^a^	4.85 (4.45–5.00) ^a^	4.65 (4.40–4.90) ^a^
HGB [g/dL]	10.10 (9.25–10.35) ^a^	12.30 (11.95–13.55) ^b^	10.20 (9.28–10.48) ^a^	13.30 (12.70–13.75) ^b^	10.20 (9.60–10.80) ^a^	13.05 (12.30–13.70) ^b^
HCT [%]	33.90 (33.80–34.00) ^a,b^	38.40 (37.15–40.50) ^b^	32.75 (30.43–33.43) ^a^	40.00 (37.65–41.00) ^b^	32.75 (32.60–33.55) ^a^	38.50 (37.00–41.20) ^b^
MCV [µm^3^]	71.00 (61.00–72.50) ^a^	80.00 (79.00–84.00) ^b^	67.00 (62.75–72.00) ^a^	82.00 (80.00–87.00) ^b^	69.50 (66.25–77.00) ^a^	83.50 (81.00–86.75) ^b^
MCH [pg]	21.80 (18.55–22.10) ^a^	26.40 (26.05–27.00) ^b^	20.40 (19.68–22.38) ^a^	27.80 (26.90–28.80) ^b^	21.75 (20.10–23.30) ^a^	27.80 (27.10–28.70) ^b^
MCHC [g/dL]	30.55 (30.08–31.03) ^a^	32.80 (32.25–33.20) ^a^	31.30 (30.20–31.65) ^a^	33.20 (32.80–33.90) ^b^	30.95 (30.10–31.83) ^a^	33.30 (32.70–34.00) ^b^
RDW [%]	17.95 (17.83–18.08) ^b^	12.50 (12.40–12.90) ^a,b^	16.60 (15.98–16.85) ^b^	13.10 (12.53–13.65) ^a,b^	15.30 (13.78–15.80) ^b^	13.00 (12.40–13.55) ^a^
Fe [µg/dL]	56.00 (40.00–75.50) ^b^	71.50 (65.25–77.75) ^b^	23.00 (20.50–27.75) ^a^	105.00 (52.00–114.00) ^b^	36.00 (15.75–75.00) ^b^	56.50 (51.75–91.25) ^b^
TTG [U/mL]	405.6 (331.8–479.3) ^a^	321.0 (135.9–396.0) ^a^	301.0 (143.7–801.0) ^a^	194.5 (34.50–801.0) ^a^	240.5 (104.5–456.5) ^a^	161.0 (39.15–733.5) ^a^

^1^ Comparison between the groups using Kruskal–Wallis analysis of variance. Values with the same letter in each row do not differ significantly (*p* < 0.05). RBC—red blood cells; HGB—hemoglobin; HCT—hematocrit; MCV—mean corpuscular volume; MCH—mean corpuscular hemoglobin; MCHC—mean corpuscular hemoglobin concentration; RDW—red cell distribution width; Fe—iron; TTG—auto-antibodies directed against tissue transglutaminase 2.

**Table 3:** Hematological characteristics in children with CD after 12 months of GFD, according to the genotype. Data are expressed as a median (P25–P75).

Parameter	TT Homozygote		CC Homozygote		CT Heterozygote	
N	10		40		56	
RBC [×10^6^/mm^3^]	4.65 (4.60–4.93) ^a 1^		4.70 (4.50–5.00) ^a^		4.60 (4.35–4.80) ^a^	
HGB [g/dL]	12.80 (11.90–13.20) ^a,b^		13.30 (12.63–14.13) ^b^ **		12.60 (12.00–13.45) ^a^ **	
HCT [%]	38.60 (37.20–39.53) ^a,b^		40.15 (37.88–41.35) ^b^ **		37.80 (36.40–40.55) ^a^	
MCV [µm^3^]	82.00 (75.00–84.00) ^a^ *		84.00 (80.75–87.00) ^a^ **		84.00 (80.75–86.25) ^a^	
MCH [pg]	27.00 (26.10–27.60) ^a^		27.85 (27.15–29.38) ^a^ ***		28.30 (26.85–29.07) ^a^ **	
MCHC [g/dL]	33.40 (32.53–34.00) ^a^ *		33.35 (32.60–33.75) ^a^		33.40 (33.00–33.60) ^a^ **	
RDW [%]	13.20 (12.30–14.80) ^a^		12.90 (12.38–13.65) ^a^		12.90 (12.55–13.75) ^a^	
Fe [µg/dL]	75.00 (57.75–107.8) ^a^		98.50 (63.00–118.5) ^a^		69.00 (45.25–96.75) ^a^	
Ferritin [ng/mL]	16.60 (13.00–33.40) ^a^		18.00 (8.10–29.25) ^a^		17.95 (10.23–38.70) ^a^	
TTG [U/mL]	3.00 (0.75–33.00) ^a^ **		5.60 (1.33–14.70) ^a^ **		4.75 (1.48–18.28) ^a^ ***	
Hepcidin [ng/dL]	219.7 (210.1–275.5) ^a^		219.6 (208.7–293.5) ^a^		234.6 (208.4–285.7) ^a^	
	Anemic ^2^	Non-anemic ^2^	Anemic	Non-anemic	Anemic	Non-anemic
N	3	7	8	32	14	42
RBC [× 10^6^/mm^3^]	4.70 (4.60–4.80) ^a^	4.60 (4.605–5.00) ^a^	4.70 (4.58–5.10) ^a^	4.80 (4.58–5.03) ^a^	4.50 (4.28–4.80 ^a^	4.60 (4.40–4.90) ^a^
HGB [g/dL]	12.40 (11.00–12.70) ^a,b^ **	12.80 (11.90–13.40) ^a,b^	12.50 (12.08–13.20) ^a,b^ **	13.65 (12.98–14.30) ^b^ **	12.05 (11.78–12.73) ^a^ ***	13.10 (12.45–13.60) ^a,b^
HCT [%]	39.35 (38.80–39.90) ^a,b^ *	38.40 (36.00–39.40) ^a,b^	37.95 (37.08–40.13) ^a,b^ ***	41.20 (39.20–42.43) ^b^ *	36.55 (35.63–38.08) ^a^ ***	38.70 (37.00–41.10) ^a,b^
MCV [µm^3^]	83.00 (70.00–84.00) ^a^ *	82.00 (79.00–84.00) ^a^	79.00 (75.00–83.00) ^a^ *	84.00 (82.00–87.25) ^a^ *	81.50 (78.00–85.00) ^a^ **	85.00 (81.00–87.00) ^a^
MCH [pg]	25.80 (23.00–27.60) ^a^	27.20 (26.40–28.30) ^a^	26.25 (24.88–27.55) ^a^ *	28.00 (27.40–29.63) ^a^ **	26.92 (25.50–28.38) ^a^ **	28.45 (27.48–29.18) ^a^
MCHC [g/dL]	31.90 (31.10–32.70) ^a^	33.50 (33.00–34.00) ^a^ *	33.00 (32.38–33.63) ^a^	33.50 (32.75–34.13) ^a^	33.20 (32.98–33.35) ^a^ **	33.40 (33.20–33.60) ^a^
RDW [%]	13.65 (11.90–15.40) ^a^	13.20 (12.60–14.20) ^a^	13.30 (12.28–14.83) ^a^ *	12.85 (12.40–13.65) ^a^ *	13.50 (12.68–14.43) ^a^	12.90 (12.40–13.50) ^a^
Fe [µg/dL]	68.00 (65.00–71.00) ^a^	106.0 (36.00–113.0) ^a^	64.00 (55.50–71.00) ^a^	105.0 (58.00–123.0) ^a^	63.00 (36.75–77.50) ^a^	69.50 (53.00–103.3) ^a^
Ferritin [ng/mL]	13.60 (10.90–16.60) ^a^	21.20 (13.00–33.40) ^a^	17.25 (16.50–18.00) ^a^	23.75 (7.95–30.18) ^a^	29.70 (2.60–39.80) ^a^	17.50 (12.00–37.90) ^a^
TTG [U/mL]	46.50 (9.30–83.70) ^a^ *	2.90 (0.8–16.10) ^a^ *	11.85 (1.98–14.70) ^a^ *	3.10 (1.0–11.90) ^a^ ***	5.20 (1.05–33.05) ^a^ **	3.60 (1.57–19.15) ^a^ ***

^1^ Comparison between the groups using Kruskal–Wallis analysis of variance. Values with the same letter in each row do not differ significantly (*p* < 0.05). ^2^ Classified at diagnosis. *, **, *** Significant differences comparing the results in the moment of diagnosis and after 12 months analyzed using Student’s t-test for dependent variables or Wilcoxon signed-rank tests, as appropriate according to the normality. (*) = *p*-value < 0.05; (**) = *p*-value < 0.01; (***) = *p*-value < 0.001. RBC—red blood cells; HGB—hemoglobin; HCT—hematocrit; MCV—mean corpuscular volume; MCH—mean corpuscular hemoglobin; MCHC—mean corpuscular hemoglobin concentration; RDW—red cell distribution width; Fe—iron; TTG—auto-antibodies directed against tissue transglutaminase 2.

## Data Availability

Data are contained within the article or Supplementary Material.

## Supplementary Materials

The following are available online at https://www.mdpi.com/article/10.3390/nu13082782/s1, Table S1. Hemoglobin levels to diagnose anemia [g/dL] (WHO recommendation).
