# Supplementary material for: TMPRSS6 rs855791 Polymorphism Status in Children with Celiac Disease and Anemia
Source: Nutrients. 2021 Aug 13;13(8):2782. doi: 10.3390/nu13082782 (PMC8398390; doi:10.3390/nu13082782)
Supplement: Supplementary file 1 [file nutrients-13-02782-s001.zip › nutrients-1314204-SI.pdf]

Supplementary Table S1. Haemoglobin levels to diagnose anemia [g/dL] (WHO recommendation) [1].

Population	Non-Anaemia	Anaemia		
		Mild	Moderate	Severe
Children 6–59 months of age	≥11.0	10.0–10.9	7.0–9.9	<7.0
Children 5–11 years of age	≥11.5	11.0–11.4	8.0–10.9	<8.0
Children 12–14 years of age	≥12.0	11.0–11.9	8.0–10.9	<8.0
Non-pregnant woman (15 years of age and above)	≥12.0	11.0–11.9	8.0–10.9	<8.0
Men (15 years of age and above)	≥13.0	11.0–12.9	8.0–10.9	<8.0

1. WHO. *Haemoglobin Concentrations for the Diagnosis of Anaemia and Assessment of Severity*; World Health Organization: Geneva, Switzerland, 2011; pp. 1–6.
